# Supplementary material for: Learning curve and surgical outcome of robotic assisted colorectal surgery with ERAS program
Source: Sci Rep. 2022 Nov 29;12:20566. doi: 10.1038/s41598-022-24665-w (PMC9709162; doi:10.1038/s41598-022-24665-w)
Supplement: Supplementary file 1 — Supplementary Information 1. [file 41598_2022_24665_MOESM1_ESM.zip › LSC Raw data-Ñ[▒K/124/N_ñw╝╨░O▒Kñσ.pdf]

W55 / W55

臺中榮民總醫院
手術室護理記錄

[redacted]

基本資料

- 室別： 二醫手術室-31室，手術科別： CRS，手術類別： 常規，日期： 2021/04/14
- 進入時間： 0750 ； 麻醉開始時間： 0800 ； Time Out時間： 0900 ； 劃刀時間： 0902 ； 手術結束時間： 1150 送出時間： 1155 ；
- 麻醉方式： GE
  診斷：Malignant tumor of sigmoid colon
  手術名稱：Laparoscopic Anterior resection and anastomosis - malignant
  手術局部用藥：NIL
  術前預防性抗生素：
  - 抗生素名稱一： Veterin 2GM IVD由麻醉護理師給予
  - 時間： 0855
  - 上次簽章: 黃鈺琇

  ，追加預防性抗生素：
- 手術醫師： 林俊余
- 麻醉醫師： VS 賴慧卿
-
  - 刷手護士A ： 李庭，時間： 0750
  - 流動護士A ： 黃鈺琇，時間： 0750

皮膚狀況

A-V Shunt： 無
術前： 完整
記錄者： 上次簽章: 黃鈺琇
術後： 完整
記錄者： 黃鈺琇

手術準備

手術臥式 單一手術臥式

-
  - 手術臥式： 截石臥式
  - 使用附件： 溫毯 38 ℃，頭圈
  - 皮膚準備： 10%Aq Betadine，Hibiscrub，2% Chlorhexidine
  - 電燒： 使用雙極電燒，記錄者： 上次簽章: 黃鈺琇

止血帶T： 無

植入物

- 植入物名稱/號碼/數量/部位：Hyalobarrigel 40mg/10ml/骨盆腔

，記錄者：上次簽章: 黃鈺琇

標本送檢

- 細菌培養 無
- 細胞學 無
- 冰凍切片 無
- 病理標本
  組織名稱一：s-colon
  ,
  組織名稱二：IMA root
  ,
  組織名稱三：distal cutend
- 截肢 無

標本交付 標本： 壹 袋，標本： 零 盒，標本： 貳 瓶，細胞學： 零 管，Culture Tube： 零 管，被交付醫師： CR 楊曙榮，

- 記錄者： 黃鈺琇
- 時間： 1155

傷口術後敷料

傷口敷料： 傷口敷料： 無

- 請於圖上標示部位：

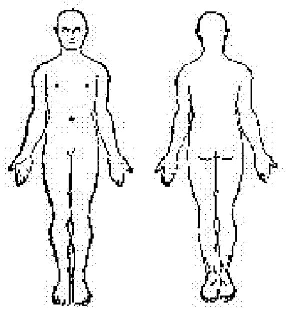

腹部腹腔鏡肆切口

- 傷口敷料： mepore

導管

導管	天數	導管資訊	插入日期	插入時間	更換日期	拔除日期	拔除時間	拔除原因	預計拔除/更換	預計拔除/更換日期
Foley(1)	1	號碼:16 Fr.; 部位:尿道;	2021/04/14	0835						
Double Lumen CVC(1)	1	號碼:8 Fr.; 部位:; 象限:RU; 註記:fix:15cm;	2021/04/14	0825						
Sump NG Tube(1)	1	號碼:16 Fr.; 部位:胃; 註記:fix:60cm;	2021/04/14	0835						
A-line (1)	1	號碼:20 號; 部位:左手橈動脈;	2021/04/14	0820						

用物計數

- 手術中出血量： minimal c.c.
- 手術中尿量： 300 c.c.
-
    - 其它： 無

用物計數： 多切口同時縫合

- 1.器械： 正確，2.縫針： 正確，3.刀片： 正確，4.紗布： 正確，5.腹部墊： 未使用，6.紗球： 未使用，7.PEANUTS ： 未使用，8.紗條： 未使用，9.絲帶： 未使用，10.針頭： 未使用，11.Vessel loop： 未使用，12.Boots： 未使用，13.Jelco： 未使用，14.棉條： 未使用，
    - 第一次：
        - 刷手護士 ： 李庭
        - 流動護士： 上次簽章: 黃鈺琇
        - 時間： 1050

  ,
    - 第二次：
        - 刷手護士 ： 李庭
        - 流動護士： 上次簽章: 黃鈺琇
        - 時間： 1115

  ,
    - 第三次：
        - 刷手護士 ： 李庭
        - 流動護士： 上次簽章: 黃鈺琇
        - 時間： 1120

備註

備註：NIL

- 記錄者： 黃鈺琇
- 時間： 1155

填寫護理人員 黃鈺琇

正式摘要

W55 / W55

doctorRank:B
